# Society Notes

**Published:** 1897-08

**Authors:** W. H. Monday

**Affiliations:** President; Terrell, Texas


### Society Notes.

TERRELL, TEXAS, June 15, 1897.

DEAR DOCTOR:—The fifth meeting of the Texas Association of Railway Surgeons will be held in the city of Galveston on the second Tuesday in August. As that is a very desirable time to visit the "Island City," and being solicitous of having the

Association well represented by the Railway Surgeons of the State, let me insist that you attend, and if feasible, bring a short practical paper on some subject pertaining to Railway Surgery, to be read before the Association.

Come, let us meet, discuss surgical questions and reason together in behalf of our profession, thereby promoting the surgical interest of the railways we represent.

A nice programme will be provided for the entertainment of the Association.

The title of all papers should be sent to Dr. Clay Johnson, Secretary, at Corsicana, Texas, not later than July 20th, in order that the programme may be issued.

I hereby extend to you a cordial invitation, insuring you a pleasant and profitable time.

For any desired information apply to Secretary Dr. Clay Johnson, or to

Yours fraternally,

W. H. MONDAY, M. D., President.

